# Undesirable Consequences of Insecticide Resistance following *Aedes aegypti* Control Activities Due to a Dengue Outbreak

**DOI:** 10.1371/journal.pone.0092424

**Published:** 2014-03-27

**Authors:** Rafael Maciel-de-Freitas, Fernando Campos Avendanho, Rosangela Santos, Gabriel Sylvestre, Simone Costa Araújo, José Bento Pereira Lima, Ademir Jesus Martins, Giovanini Evelim Coelho, Denise Valle

**Affiliations:** 1 Laboratório de Transmissores de Hematozoários, Instituto Oswaldo Cruz, Fiocruz, Rio de Janeiro, Brazil; 2 Secretaria de Vigilância em Saúde, Ministério da Saúde, Brasília, Brazil; 3 Secretaria Estadual de Saúde de Roraima, Boa Vista, Brazil; 4 Laboratório de Fisiologia e Controle de Artrópodes Vetores, Instituto Oswaldo Cruz, Fiocruz, Rio de Janeiro, Brazil; 5 Instituto de Biologia do Exército, Rio de Janeiro, Brazil; 6 Instituto Nacional de Ciência e Tecnologia em Entomologia Molecular (INCT-EM), Rio de Janeiro, Brazil; 7 Laboratório de Biologia Molecular de Flavivírus, Instituto Oswaldo Cruz, Fiocruz, Rio de Janeiro, Brazil; Institut Pasteur, France

## Abstract

**Background:**

During a dengue outbreak with co-circulation of DENV-1 and -2 in the city of Boa Vista, one patient was diagnosed with DENV-4, a serotype supposed absent from Brazil for almost 30 years. The re-emergence of DENV-4 triggered the intensification of mechanical and chemical *Aedes aegypti* control activities in order to reduce vector density and avoid DENV-4 dissemination throughout the country.

**Methods/Principal Findings:**

Vector control activities consisted of (a) source reduction, (b) application of diflubenzuron against larvae and (c) vehicle-mounted space spraying of 2% deltamethrin to eliminate adults. Control activity efficacy was monitored by comparing the infestation levels and the number of eggs collected in ovitraps before and after interventions, performed in 22 Boa Vista districts, covering an area of ∼80% of the city and encompassing 56,837 dwellings. A total of 94,325 containers were eliminated or treated with diflubenzuron. The most frequently positive containers were small miscellaneous receptacles, which corresponded to 59% of all positive breeding sites. Insecticide resistance to deltamethrin was assessed before, during and after interventions by dose-response bioassays adopting WHO-based protocols. The intense use of the pyrethroid increased fourfold the resistance ratio of the local *Ae. aegypti* population only six months after the beginning of vector control. Curiously, this trend was also observed in the districts in which no deltamethrin was applied by the public health services. On the other hand, changes in the resistance ratio to the organophosphate temephos seemed less influenced by insecticide in Boa Vista.

**Conclusions:**

Despite the intense effort, mosquito infestation levels were only slightly reduced. Besides, the median number of eggs in ovitraps remained unaltered after control activity intensification. The great and rapid increase in pyrethroid resistance levels of natural *Ae. aegypti* populations is discussed in the context of both public and domestic intensification of chemical control due to a dengue outbreak.

## Introduction

Dengue fever is a mosquito-borne disease caused by antigenically related but distinct virus serotypes (DENV-1, -2, -3 or -4). The World Health Organization (WHO) estimates 50 million cases of dengue fever occur annually, with more than 2.5 billion people living in risk areas [1]. Dengue viruses are overwhelmingly transmitted to man through the bite of dengue bearing female *Aedes* mosquitoes. The main dengue vector worldwide is the *Ae. aegypti* mosquito, which is well adapted to urbanized areas, especially peridomestic, breeds often in man-made containers and almost exclusively bites man [2]–[5].

In spite of remarkable efforts from public health authorities, organizations and research institutes, it has proven very challenging to achieve effective and sustainable control of *Ae. aegypti* on a long-term basis and consequently, disrupt dengue outbreaks. This issue has multiple causes, including (a) uncontrolled and unplanned urbanization, providing abundant mosquito breeding sites, (b) lack of basic sanitation and regular distribution of piped water, leading to improvised storage by householders, (c) greater mobility of potentially infected hosts, increasing areas with circulation of multiple serotypes, (d) low investment in proactive vector control measures as well as in area-wide community-based approaches and (e) persistent use of vector control methods that have limited efficacy [6]–[8]. Nowadays, since there are no effective vaccines against dengue, the most recommended strategy to minimize DENV transmission relies on actions against the mosquito vector, maintaining *Ae. aegypti* abundance below a critical threshold. In order to accomplish this, many different approaches may be incorporated such as mechanical, chemical and biological control tools as well as community-engagement based programs, each with its own pros and cons [9]. In spite of all alternatives, insecticides are still a key component for the control of natural mosquito populations.

In Brazil, *Ae. aegypti* control is increasingly based on community participation campaigns that seek to fortify the importance of mechanical control through the adequate coverage/elimination of potential breading sites. Thousands of health agents are responsible for visiting residences and orienting occupants concerning good practices related to dengue prevention. Ideally, treatment with larvicides takes place in 4–6 annual cycles as a complementary measure, only on water reservoirs that cannot be eliminated. Two different larvicides are currently employed in the country, the organophosphate temephos and the chitin synthesis inhibitor diflubenzuron, the latter mainly in localities with confirmed resistance to the organophosphate. Adult mosquito control, performed by vehicle-mounted ultra-low volume (ULV) space spraying of pyrethroids, is theoretically restricted to epidemic seasons, during dengue outbreaks. However, the domestic use of pyrethroids is massive, both through aerosol cans by householders or even space spraying services hired privately by residential complexes [10].

Intense insecticide use poses a severe selection pressure on mosquitoes, favoring the increase of resistance alleles in natural populations. Resistance renders a great selective advantage in environments under insecticide selection pressure. As a consequence, insecticide resistance alleles generally increase very quickly in frequency in natural mosquito populations exposed to intense insecticide application [11]–[13]. For instance, in Mexico, after 6–8 years of permethrin overuse, a significant rise of a mutation at position 1,016 of the voltage-gated sodium channel gene was observed. This mutation, which encodes an isoleucine rather than a valine, bestows knockdown resistance to homozygous mosquitoes, impairing chemical control effectiveness over time [12]. However, to our knowledge, very rapid changes in pyrethroid resistance levels due to a dengue outbreak and the consequent intensification of vector control activities remain to be accompanied. Therefore, in this study we assess the potential changes in *Ae. aegypti* insecticide resistance rate to pyrethroids and organophosphates after intense mechanical and chemical vector control interventions, both triggered by the confirmation of a DENV-4 outbreak in the city of Boa Vista.

## Methods

### Boa Vista

The city of Boa Vista (02°49’10’’ N 60°40’16’’ W), Capital of the State of Roraima, population around 284,000, is situated on the bank of the Branco River, 220 km from the border between Brazil and Venezuela. Boa Vista has an equatorial climate, with high temperatures throughout the year (around 30°C) and a very wet environment with annual precipitation often above 2,000 mm, concentrated between April and September.

### Dengue in Boa Vista

**Figure 1:**
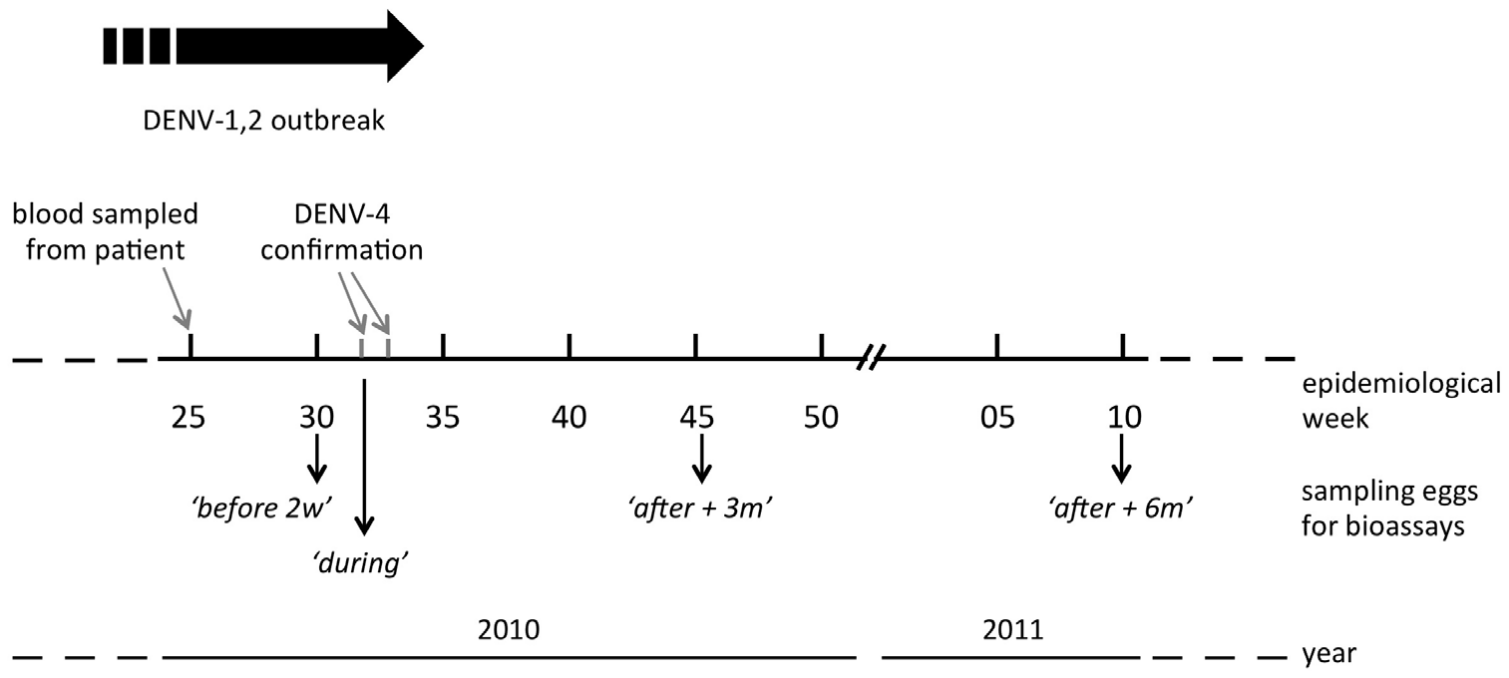
Timeline of vector control activities related to DENV-4 confirmation in Boa Vista, Roraima State. Intensification of vector source reduction activities started at the 32^nd^ epidemiological week ('*during*').

Boa Vista registered 4,226 DENV-1 and 2 co-circulating cases during 2010, a 288% increase over 2009, of which 1,567 (37.1%) were laboratory confirmed and 2,659 (62.9%) were clinical. On the 25^th^ epidemiological week (June 20^th^ to 26^th^) the blood of one patient with dengue symptoms was collected and, seven weeks later, a DENV-4 infection was confirmed after isolation from C6/36 cell samples and RT-PCR [14]. According to the Brazilian Health Ministry, this was the first notification of DENV-4 in the country after almost 30 years of absence. Two samples collected four weeks after this putative index DENV-4 case were also confirmed at the 33^th^ epidemiological week. Intensification of vector control activities started at the 32^nd^ epidemiological week, immediately after laboratory confirmation of the first case (see Figure 1 for further details).

### Vector surveillance and control routine in Boa Vista

In Brazil, *Ae. aegypti* routine surveillance is based on a strategy called Larval Index Rapid *Aedes* assay (LIRAa). It consists of a two-stage random sampling procedure with blocks of houses as the primary sampling unit and individual residences as the secondary sampling unit. A relatively small number of dwellings (a maximum of 450 out of 9,000–12,000) per district is sampled 4–6 times yearly [15]. The outcome of LIRAa is to provide House (HI) and Breteau (BI) Indexes, that reflect, respectively, the rate of positive houses and the number of breeding sites per total number of inspected houses in each district. According to recommendations of the Brazilian Dengue Control Program, this index guides vector control actions [16].

### Interventions

**Figure 2:**
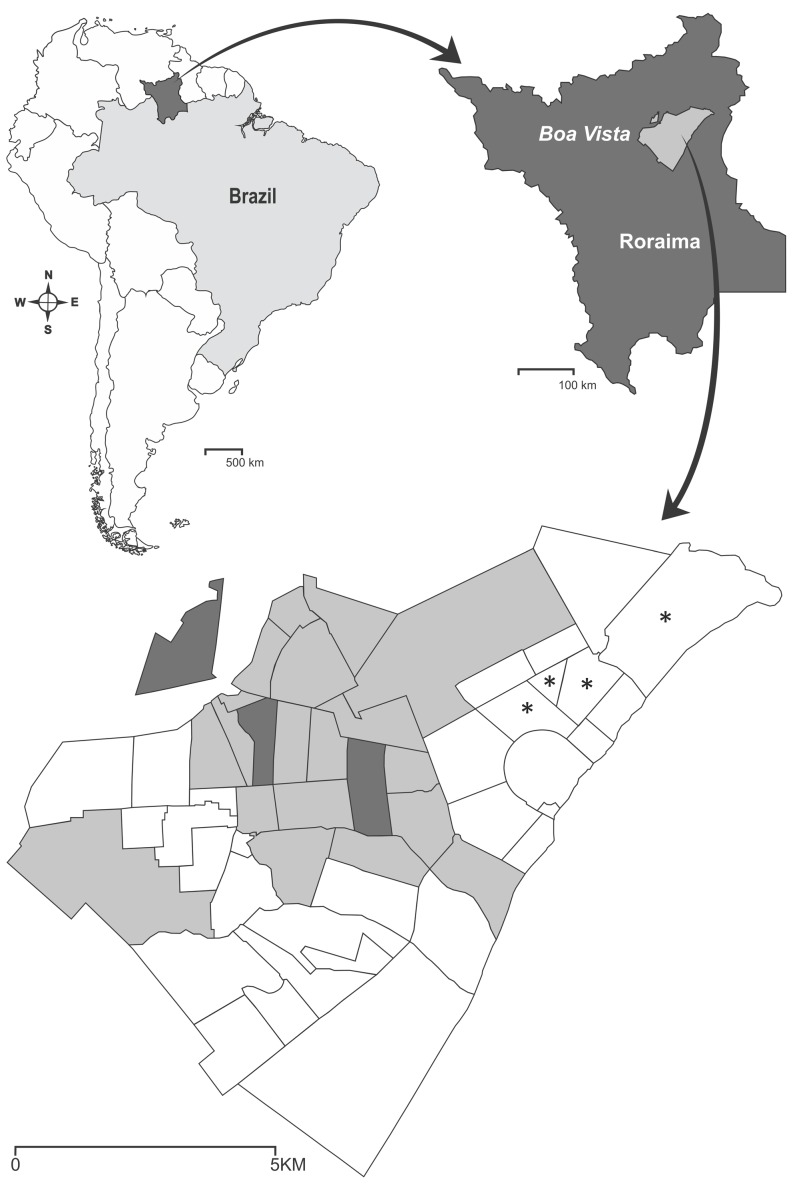
Schematic view of Boa Vista city. Districts in dark grey belong to Zone 1 and were exposed to an intense vector control, which included source reduction, diflubenzuron application against larvae and six cycles of deltamethrin applied on a vehicle-mounted basis to reduce adult mosquito density. Light grey shows Zone 2, where source reduction and diflubenzuron also took place, but only three cycles of deltamethrin were applied. Finally, Zone-3 represents all the districts that had no intervention, i.e., vectorial control was routinely performed. Insecticide resistance evaluations were performed with two groups of pooled samples: those derived from all districts of Zone 1 and, for Zone 3, material collected at districts labeled with an asterisk.

Intensification of control activities in Boa Vista started immediately after DENV-4 laboratory confirmation, as an attempt to reduce vector population and then to halt DENV-4 transmission. First, intensified interventions were restricted to the three districts where the first DENV-4 cases had been notified (the geographic area of these three districts will be called Zone 1 from now on). However, new DENV-4 cases were soon confirmed in 10 other districts, presenting a rapid spread of this dengue serotype in the city [14]. Two weeks after the beginning of interventions, control intensification was expanded to 19 additional Boa Vista districts, referred to as Zone 2, that rendered confirmed DENV-4 cases or an intense dengue transmission history. Finally, Zone 3 designates districts where no specific intensified intervention was conducted, i.e., just routine control was maintained (Figure 2). Intensified interventions were based on mechanical and chemical control measures. Mechanical control consisted of source reduction, whereas chemical control was based on the application of the insect growth regulator diflubenzuron in non-eliminated containers and on vehicle-mounted spatial spraying of pyrethroid deltamethrin against adults.

### Source reduction and infestation levels

*Ae. aegypti* source reduction was tentatively carried out in all homes of 22 districts of Boa Vista, i.e. Zones 1 and 2 (Figure 2), this area comprising around 75% of all habitations in the municipality. Upon householders' authorization, health agents inspected all containers for immature *Ae. aegypti*. Positive containers were classified into seven categories according to recommendations of the Brazilian Dengue Control Program: A1 - large water holding containers above ground level, such as cisterns, A2 - large water holding containers at ground level, such as pools or cement tanks, B - mobile containers, like metal drums and planters, pots and vases, C - fixed containers such as roof gutters, D1 – tires, D2 - miscellaneous, like domestic garbage cans and E - natural containers, such as bromeliads. When a positive container was identified, a larvae sample was collected and brought to the laboratory for further assessment, producing the HI and BI for each district. Whenever possible, potential breeding sites were removed. Permanent containers were treated with diflubenzuron, a chitin synthesis inhibitor (see below).

Fifteen days after the beginning of intensified interventions, a larval survey was conducted in all three districts of Zone 1 plus 12 of the 19 districts of Zone 2, for which, we used the rationale involved in the LIRAa sampling. Our aim was to assess the infestation levels subsequent to the intensification of control activities. In this 2^nd^ survey, a sample of approximately 10% of dwellings per district was inspected.

### Chemical control

Chemical control of larvae was performed concomitantly with source reduction in all districts of Zones 1 and 2. Permanent containers were treated with diflubenzuron, a chitin synthesis inhibitor, at a dosage of 0.25 mg/liter. Chemical control of adult *Ae. aegypti* was based on vehicle-mounted ultra-low-volume (ULV) spray applications of 2% deltamethrin at a dosage of 0.6 mg active ingredient per hectare [17]. Each round of ULV consisted of three applications, at 3–5 day intervals, as recommended by the Brazilian Guidelines for Prevention and Control of Dengue [18]. One round of vehicle-mounted ULV (three applications) was initially applied to Zone 1. However, two weeks later, due to the rapid dissemination of DENV-4 in Boa Vista, ULV application was expanded to Zone 2, two rounds of deltamethrin being employed (corresponding to six applications) in Zone 1 and one round (three applications) in Zone 2. Chemical adult control was not used in Zone 3.

### Evaluation of efficacy of control activities

Two indirect measures were adopted to evaluate whether the control activities were able to reduce mosquito density. First, we compared the infestation levels in Zones 1 and 2 during source reduction and those of the larval survey that took place two weeks later. Afterwards, we assessed the average number of eggs collected in ovitraps baited with hay infusion, which were routinely installed at weekly intervals before the Boa Vista outbreak. A total of 275 ovitraps were installed in 24 districts, comprising 50 in Zone 1, 85 in Zone 2 and 140 in Zone 3. Then, we were able to monitor the average number of eggs collected and to recognize potential changes in Zones 1 and 2, where intensified interventions took place, compared to Zone 3, where only routine activities were performed. We used the non-parametric Mann-Whitney test to evaluate whether the numbers of eggs collected per ovitrap during the five weeks precedent to the intervention actions in the three zones were different from those in the subsequent five weeks.

### Mosquito insecticide resistance monitoring

We monitored potential changes in *Ae. aegypti* insecticide resistance on a temporal basis, i.e., before, during and after intervention. The time interval “before-2w” was with eggs collected in the field two weeks prior to the beginning of interventions, the “during” with eggs collected in the same week of source reduction, diflubenzuron application and vehicle-mounted ULV in Boa Vista and the “after” (after+3 m) with eggs collected three months after intervention and “after+6 m” with *Ae. aegypti* eggs collected six months after the intervention. In all cases, eggs were obtained from ovitraps installed simultaneously in Zones 1 and 3, the districts with six cycles of vehicle-mounted ULV application and in the areas where no insecticide was applied, respectively. Specimens used for the bioassays derived from 50 ovitraps installed in the three districts comprising Zone 1 and from 40 ovitraps covering four adjacent districts at Zone 3 (Figure 2). These sample sizes were in accordance to recommendations of the Brazilian *Ae. aegypti* Insecticide Resistance Monitoring Network (Rede MoReNAa). Rearing of parental adult mosquitoes, obtaining F1 eggs and accomplishment of bioassays were also performed according to MoReNAa standard protocols [19]: L3 larvae and adult females of the F1 generation were exposed to dose-response bioassays with temephos (PESTANAL; Sigma-Aldrich) and deltamethrin (PESTANAL; Sigma-Aldrich), respectively, as recommended by WHO [20], [21]. Adult quantitative bioassays were conducted with deltamethrin impregnated papers, a method adapted from the WHO protocol.^21^ Results of at least three assays per mosquito sample on different days are shown. Lethal concentrations (LC) were calculated with Probit analysis [software Polo-PC, LeOra Software, Berkeley, CA] [22]. Resistance Ratios (RR_50_ and RR_90_) were obtained by comparison with LC values for Rockefeller, a reference strain for insecticide susceptibility and vigor [23]. In Brazil, temephos RR values above 3.0 are already considered indicative of resistance and elicit recommendation of substitution of the insecticide class employed in *Ae. aegypti* control [19].

### Ethical considerations

Before the inspection, each resident received full explanation regarding the project by the same health agent they were used to receiving in their home 4–6 times yearly during the surveillance routine. This explanation pointed out the relevance and objectives of the house inspection. Only after receiving oral consent from home owners, which was not documented, health agents entered the dwellings to perform their activity that consisted of the search and elimination of potential breeding sites or treating containers with diflubenzuron. We have not requested approval from local ethical committees because no personal information was obtained from householders and, most important, vector control was performed with the health agents of Boa Vista, which carry out house inspections on their routine following the guidelines of the Brazilian Dengue Control Program.

## Results

### Infestation index

**Figure 3:**
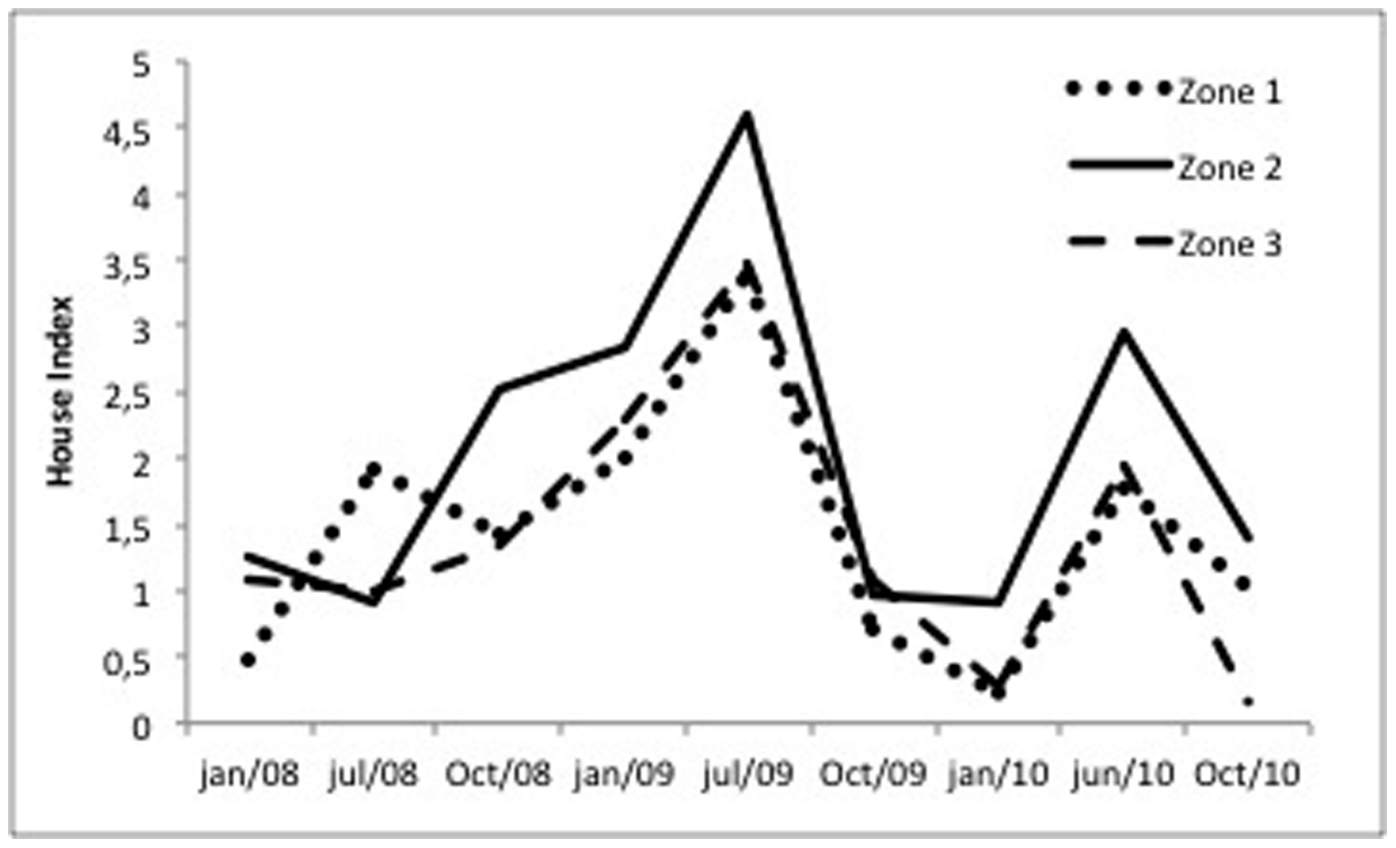
Percentage of houses harboring *Ae. aegypti* immature forms (HI) in Boa Vista between Jan/2008 and Oct/2010. Zones 1–3 are shown in Figure 2.

The HI of Boa Vista between 2008 and Jul/2010, when the DENV-1 and -2 outbreak started, was in general remarkably low. Except for the amazing values attained at July 2009, typically, less than 2.0–2.5% of the inspected houses had mosquito larvae, a pattern for Zones 1, 2 and 3 (Figure 3). In June/2010, at the peak of the DENV transmission with more than 500 confirmed cases per week, the HI varied between approximately 1.8 and 3.0 throughout the city (Figure 3). It is of note that HI attained at October 2010, after all the intervention work following DENV-4 detection, did not decrease significantly relative to the same period at previous years.

### Source reduction

Health agents inspected 56,837 homes, corresponding to 74% of all habitations in Boa Vista, and removed a total of 94,325 containers in the 22 monitored districts (Table 1). *Ae. aegypti* immature mosquitoes were collected in 968 houses, producing an overall HI of 1.70 and a BI of 1.79 (Table 1). We observed that 59.1% (n = 13) of the 22 districts where source reduction took place had an HI below 1.0 and just two districts presented an HI higher than 3.0 (data not shown). The second survey presented a slight and non-significant decrease in infestation levels, since the HI varied from 1.70 to 1.37, meanwhile the BI varied from 1.79 to 1.51 (HI: χ^2^ = 1.22, P>0.05; BI: χ^2^ = 0.88, P>0.05). Unexpectedly, three of the 15 districts exhibited an increase of both HI and BI indexes after intensified interventions, one in Zone 1 and two in Zone 2 (data not shown), probably responsible for the average low decrease for the Boa Vista infestation level.

**Table 1:** Mechanical control activities conducted in Boa Vista based on *Ae. aegypti* source reduction.

	Source reduction after visiting 100% of houses of Boa Vista												*Ae. aegypti* infestation level after sampling 10% of houses 15 days after the 1^st^ survey			
Zone of Boa Vista city	Inspected houses	Eliminated containers	Positive houses	Container classification							HI	BI	Inspected houses	Positive houses	HI	BI
				A1	A2	B	C	D1	D2	E						
Zone 1	10,358	18,305	192	2	24	30	6	34	121	8	1.85	2.17	1,205	20	1.65	2.18
Zone 2	46,479	76,020	776	13	66	87	34	93	480	19	1.67	1.70	3,753	48	1.27	1.52
Total	56,837	94,325	968	15	90	117	40	127	601	27	1.70	1.79	4,958	68	1.37	1.51

A first survey consisted of inspecting 100% of the houses in 22 districts that belonged to Zones 1 and 2. Fifteen days later, a new larval survey was conducted in 15 of these districts, sampling around 10% of houses per district.

Positive containers in Zones 1 and 2 were mostly classified as D2 (59%), which represents miscellaneous receptacles, often domestic garbage items that can become small isolated breeding sites. Taken into account the irregular water supply services in several Boa Vista neighborhoods, it was somewhat surprising that only 1.47% of the *A. aegypti* samples were obtained from cisterns and metal drums.

### Insecticide resistance monitoring

The intensification of vector control measures in Boa Vista resulted in a dramatic increase in the resistance status of mosquito samples to the deltamethrin pyrethroid, targeting adults (Table 2). Three months after the beginning of intensified intervention, resistance ratios of 10–15 jumped to values around 30, and after another three months, these values were even higher, about 40–50. Simultaneously, there was a significant decrease in the heterogeneity of mosquito samples as judged by the slope values of the mortality curves. It is noteworthy that mosquito populations from both sampled areas, exposed or not to government intensification control, exhibited an increase in resistance ratio and also a variability loss.

**Table 2:** Resistance status to the pyrethroid deltamethrin of Boa Vista *Ae. aegypti* adults collected as eggs at Zones 1 and 3.

Area of collection	Time interval	LC_50_ (*) (mg/m^2^)	Confidence Interval LC_50_ (*) (mg/m^2^)	RR_50_(**)	LC_90_ (*) (mg/m^2^)	Confidence Interval LC_90_(*) (mg/m^2^)	RR_90_(**)	Slope
	before-2w	7.388	6.82387 – 7.99881	10.5	19.567	17.18934 – 22.27293	15.8	3.0
	during	6.600	6.21028 – 7.01439	9.4	18.519	17.23321 – 19.90134	14.9	2.9
Zone 3	after+3 m	18.050	15.36343 – 21.20523	25.7	36.117	34.24400 – 38.09289	29.1	4.3
	after+6 m	33.323	32.74471 – 33.91077	47.4	47.177	45.76803 – 48.62923	38.1	8.5
	before-2w	10.011	9.47114 – 10.58076	14.2	19.760	17.92063 – 21.78781	15.9	4.3
	during	8.482	8.03627 – 8.95248	12.1	24.343	22.07974 – 26.83836	19.6	2.8
Zone 1	after+3 m	22.308	20.83174 – 23.88858	31.7	44.281	42.23937 – 46.42181	35.7	4.3
	after+6 m	32.299	30.59017 – 34.10248	45.9	48.130	43.74962 – 52.94873	38.8	7.4
Rockefeller		0.7031	0.65731 – 0.74531	1.0	1.239	1.16114 – 1.33965	1.0	5.2

(*) Lethal concentration.

(**) Resistance ratio.

Samples were collected two weeks before, during or after (three or six months) the intensification of control efforts, performed with both mechanical and chemical actions.

Monitoring of the temephos susceptibility status of the same samples (Table 3) confirmed the slow increase of resistance levels to this organophosphate. However, progressively higher slope values were again obtained, pointing to a significant loss of the vector populations' variability since the onset of control intensification. Similar to the response to deltamethrin, no overall differences between the two areas, Zones 1 and 3, were evident in the case of temephos resistance status.

**Table 3:** Resistance status to the organophosphate temephos of Boa Vista *Ae. aegypti* larvae collected as eggs at Zones 1 and 3.

Area of collection	Time interval	LC_50_ (*) (mg/m^2^)	Confidence Interval LC_50_ (*) (mg/m^2^)	RR_50_(**)	LC_90_ (*) (mg/m^2^)	Confidence Interval LC_90_(*) (mg/m^2^)	RR_90_(**)	Slope
	before-2w	0.00525	0.00339–0.00812	1.34	0.01587	0.00877–0.02874	2.18	3.0
	during	0.00911	0.00852–0.00973	2.33	0.01738	0.01573–0.01920	2.39	2.9
Zone 3	after+3 m	0.00936	0.00884–0.00990	2.39	0.017	0.01563–0.01849	2.34	4.3
	after+6 m	0.01116	0.01051–0.01185	2.85	0.0198	0.01771–0.02214	2.72	8.5
	before-2w	0.00792	0.00545–0.01152	2.03	0.0244	0.01289–0.04616	3.36	4.3
	during	0.01062	0.01008–0.01119	2.72	0.02164	0.01969–0.02378	2.98	2.8
Zone 1	after+3 m	0.00951	0.00898–0.01007	2.43	0.01771	0.01620–0.01937	2.44	4.3
	after+6 m	0.01839	0.01762–0.01920	4.70	0.03261	0.03070–0.03464	4.49	7.4
Rockefeller		0.00391	0.00379–0.00404	1.0	0.00727	0.00687–0.00770	1.0	5.2

(*) Lethal concentration.

(**) Resistance ratio.

Samples were collected two weeks before, during or after (three or six months) the intensification of control efforts, performed through both mechanical and chemical actions.

### Efficacy evaluation of control activities

**Figure 4:**
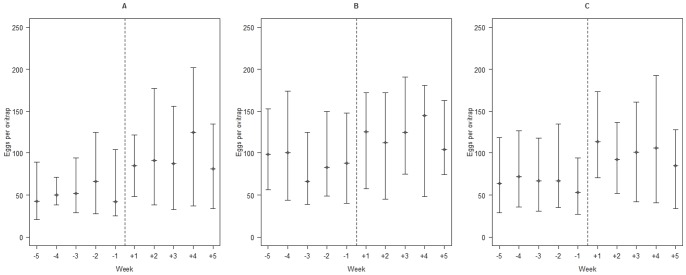
Median, 25 and 75 percentiles for the quantitative series of eggs collected in ovitraps in (A) Zone 1, (B) Zone 2 and (C) Zone 3. The dashed line refers to the intervention, and the number of eggs per ovitrap per week was assessed before (five weeks prior) and after (five weeks later) the intensification of control activities.

The impact of intensified control on vector population was measured by monitoring egg density in ovitraps. The number of eggs per ovitrap presented a remarkable non-normal and overdispersion distribution (Shapiro-Wilk normality test: Zone 1: W = 0.8619, P < 0.001; Zone 2: W = 0.901, P < 0.001; Zone 3: W = 0.901, P < 0.001). The increase in the median number of eggs per ovitrap after interventions was statistically significant in all Zones (Zone 1: U = 6090, P = 0.0003; Zone 2: U = 16610, P = 0.0045; Zone 3: U = 20900, P = 0.0001). Curiously, the general pattern of increase during the evaluated period was similar in all Zones (Figure 4).

## Discussion

In this report we demonstrated how the insecticide resistance rate of *Ae. aegypti* mosquitoes to pyrethroids and to the organophosphate temephos may be influenced by the intensification of vector chemical control. In our particular case, the intervention was due to the need of rapidly reducing mosquito population density in order to halt DENV-4 transmission. Although the exact period of DENV-4 re-entry in the country is a matter of debate, according to the Brazilian Health Ministry the detection of this serotype at Boa Vista corresponded to the first case, after an absence for almost 30 years [14], [24]. During an outbreak, dengue decision-makers must use the available tools to provide a rapid and effective response against DENV. So far, chemical compounds are one of the most popular tools in these situations. Our point is that the overuse of insecticides, even for a short period of time, poses a formidable selection pressure on natural mosquito populations, quickly selecting those insects with resistance alleles, as evidenced in Mexico [12]. The rapid selection of resistance alleles in mosquito populations is partially supported by data gathered under laboratory-controlled conditions and artificial selection. For instance, the Ile1,016 allele frequencies increased in six *Ae. aegypti* lines within only five generations of permethrin application [25]. The rapid increase of insecticide resistance alleles in *Ae. aegypti* field populations emphasizes the need for developing alternative strategies such as insecticide rotations and mixtures to delay the evolution of resistance, a phenomenon that most likely encumbers dengue control programs [26]–[28].

### Source reduction and infestation levels

Vector control routine in Brazil is not focused on pupal surveys to determine container productivity, but rather on the frequency of positive premises or containers in a given area, by means of the LIRAa strategy [15], [29], [30]. Container types that harbor pupae are not targeted in subsequent control measures, i.e., no special attention is paid to those recipients greatly contributing to the adult mosquito population. The LIRAa survey produces as its outcome HI and BI infestation indexes, but this sampling strategy has severe limitations. This is illustrated by the HI variations in equivalent seasons in the recorded period at Boa Vista (Figure 3). In addition, Pilger et al. [30] compared infestation indices and, observed that pupal surveys constantly report higher infestation levels than the LIRAa. HI and BI, as produced by LIRAa, present a limited correlation with adult mosquito populations [3], [29], [30], which may be the main reason to explain why even with low infestation levels, Boa Vista still faced a severe dengue outbreak.

### Monitoring insecticide resistance

Chemical insecticides have been widely utilized in Brazil for several years, sometimes as the primary vector control strategy at the Municipal level. Clearly, insecticide overuse poses a high selection pressure and favors the dissemination of resistance alleles in natural populations [28]. Disregarding potential technical issues on vehicle-mounted ULV application such as wind speed, drop size, etc., deltamethrin application produced low effect in reducing *Ae. aegypti* population size.

Comparisons of pyrethroid resistance status throughout and after intensified control revealed a significant increase in the resistance ratio values. This occurred in Zone 1, exposed to six vehicle-mounted ULV cycles during the chemical intervention, as well as in Zone 3, where ULV applications did not take place. Although equivalent before and during the course of the control activities, RR_50_ and RR_90_ doubled in almost all evaluated samples three months after the intervention and increased up to three-four times after an additional three months (Table 2).

In both areas, a tendency of slope increase was noted after the chemical intervention, when compared to the initial values. Six months after control, these values were even higher than Rockefeller, a known homogeneous reference *Ae. aegypti* strain, kept under laboratory conditions for decades [23]. Unexpectedly, this variability loss was greater in Zone 3, void of vehicle-mounted ULV cycles and diflubenzuron application. High levels of pyrethroid resistance, evidenced through both bioassays and molecular biology tools, have been recently reported in several Brazilian localities and are related to the intense use of this insecticide [33], [34].

In contrast to the deltamethrin resistance status increase, RR values to the larvicide temephos remained indicative of a low alteration profile during the whole evaluation period. This was an expected result, considering that, as in several other Brazilian municipalities, temephos was not applied in Boa Vista since 2001 by public health authorities. It was interesting to note that temephos RR values were much lower compared to those obtained throughout the country when this organophosphate was still the major larvicide employed against the dengue vector [19]. Together, these data corroborate the effectiveness of rotation, or temporal interruption, of insecticides towards the achievement of a rational management of resistance.

Concerning the temephos data of Boa Vista samples here presented, a consistent loss of heterogeneity of all populations throughout the evaluated period was noted, an expected outcome of the chemical control intensification. This loss of heterogeneity regarding temephos resistance, probably a by-product of pyrethroid selection pressure, also suggests a certain level of cross resistance between both classes of insecticides, reinforcing the importance of the continuous monitoring of insecticide resistance of natural *Ae. aegypti* populations.

The relevance of the domestic use of insecticides in aerosol cans, stimulated by the outbreak and re-entry of DENV-4 in the country, is still unknown, and its potential influence on pyrethroid resistance increase must not be ruled out. However, the comparable RR increase in both Zones 1 and 3 corroborates the importance of this intense domestic insecticide use towards the selection of resistant populations. Therefore, insecticide application against adults should be restricted to dengue outbreaks rather than routine practice, otherwise further chemical control may be impaired by increased insecticide resistance levels of natural vector populations.

### Efficacy of control activities

After conducting an intensification of control measures based on concomitant mechanical and chemical *Ae. aegypti* control, a dramatic reduction in mosquito density was obviously expected. However, the average number of eggs collected per week remained roughly unaltered after intervention. Furthermore, the average number of eggs collected per ovitrap was not affected: equivalent profiles being obtained for Zones 1, 2 and 3, this last one a control, not exposed to the intensification of intervention actions. Our results suggest low efficacy of the interventions carried out in Boa Vista, which may be due to several uncontrolled variables such as the low adherence of population to prevention activities and breeding sites elimination, health agent motivation, householder reluctance to health agent home inspection of potential breeding sites or keeping their windows open during ULV applications, for instance.

An alternative explanation is the effective elimination of potential mosquito breeding sites through mechanical control. Since *Ae. aegypti* mosquitoes are able to lay eggs in several different container types, elimination of highly productive recipients may increase egg laying in and the relative productivity of other containers, ovitraps possibly being included. Nevertheless, successful mechanical control is labor-intensive, costly and time-consuming. Regardless of serious efforts and scrutiny, some containers are bound to be overlooked. Likewise, pyrethroid application against adults was inhibited by the baseline high resistance status of Boa Vista *Ae. aegypti* mosquitoes.

As the main outcome of governmental interventions in Boa Vista, in August 2010, the intensification of vector control activities seemed unable to halt DENV-4 spread. Less than one year later, at March 2011, DENV-4 had been detected in nine Brazilian States [35], and in May 2012, the Ministry of Health declared DENV-4 was reported in 23 out of 27 Brazilian States. Although Souza et al. [24] speculated that DENV-4 underwent a silent circulation in the Southeast Region, detection of DENV-4 in other States started to occur soon after the Boa Vista outbreak. Nonetheless, it is important to consider the complementary character of every specific intervention event. An effective dengue control surpasses the scope of public health and involves not only adequate sanitation measures, such as regular garbage collection and water supply, but also a higher awareness of local residents and managers regarding the importance of keeping their dwellings and public spaces in proper hygienic conditions, free of potential breeding sites. In this sense, community engagement efforts towards the involvement of citizens and scholars regarding the importance of a continuous entomological surveillance should be considered of fundamental importance.
